# CBCT‐based navigation system for open liver surgery: Accurate guidance toward mobile and deformable targets with a semi‐rigid organ approximation and electromagnetic tracking of the liver

**DOI:** 10.1002/mp.14825

**Published:** 2021-04-01

**Authors:** Oleksandra V. Ivashchenko, Koert F.D. Kuhlmann, Ruben van Veen, Bas Pouw, Niels F. M. Kok, Nikie J. Hoetjes, Jasper N. Smit, Elisabeth G. Klompenhouwer, Jasper Nijkamp, Theodoor J. M. Ruers

**Affiliations:** ^1^ Department of Surgical Oncology The Netherlands Cancer Institute Antoni van Leeuwenhoek Hospital Plesmanlaan 121 1066 CX Amsterdam The Netherlands; ^2^ Department of Radiology The Netherlands Cancer Institute Antoni van Leeuwenhoek Hospital Plesmanlaan 121 1066 CX Amsterdam The Netherlands; ^3^ Faculty of Science and Technology (TNW) University of Twente Drienerlolaan 5 7522 NB Enschede The Netherlands; ^4^ Present address: Department of Radiology Leiden University Medical Center Albinusdreef 2 2333 ZA Leiden The Netherlands; ^5^ Present address: Department of Surgical Oncology The Netherlands Cancer Institute Antoni van Leeuwenhoek Hospital Plesmanlaan 121 1066 CX Amsterdam The Netherlands

**Keywords:** image guidance, liver surgery, surgical navigation, tumor tracking

## Abstract

**Purpose:**

The surgical navigation system that provides guidance throughout the surgery can facilitate safer and more radical liver resections, but such a system should also be able to handle organ motion. This work investigates the accuracy of intraoperative surgical guidance during open liver resection, with a semi‐rigid organ approximation and electromagnetic tracking of the target area.

**Methods:**

The suggested navigation technique incorporates a preoperative 3D liver model based on diagnostic 4D MRI scan, intraoperative contrast‐enhanced CBCT imaging and electromagnetic (EM) tracking of the liver surface, as well as surgical instruments, by means of six degrees‐of‐freedom micro‐EM sensors.

**Results:**

The system was evaluated during surgeries with 35 patients and resulted in an accurate and intuitive real‐time visualization of liver anatomy and tumor's location, confirmed by intraoperative checks on visible anatomical landmarks. Based on accuracy measurements verified by intraoperative CBCT, the system’s average accuracy was 4.0 ± 3.0 mm, while the total surgical delay due to navigation stayed below 20 min.

**Conclusions:**

The electromagnetic navigation system for open liver surgery developed in this work allows for accurate localization of liver lesions and critical anatomical structures surrounding the resection area, even when the liver was manipulated. However, further clinically integrating the method requires shortening the guidance‐related surgical delay, which can be achieved by shifting to faster intraoperative imaging like ultrasound. Our approach is adaptable to navigation on other mobile and deformable organs, and therefore may benefit various clinical applications.

## 1 INTRODUCTION

Annually, liver malignancies affect more than 1.4 million people worldwide, either as a result of primary liver cancer or as metastases from other cancers.^1^, ^2^ For a large fraction of these patients, liver resection remains the best treatment option with respect to patient prognosis and may even offer curation.^3^

Due to complexity of the liver anatomy, various preoperative imaging modalities (e.g., CT or MRI) are used to determine the resectability and to plan the surgical approach.^4^ This detailed plan is developed in consideration of the tumors’ locations with respect to major blood vessels and biliary anatomy.^5^, ^6^, ^7^ Despite the extensive pre‐operative resection planning, the resection itself is still primarily based on the surgeon’s recollection of the preoperative images, intraoperative tactile feedback and its correlation with live 2D ultrasound images. This lack of detailed imaging information during the procedure increases probability of intra‐ or post‐operative hepatic complications, which occur in up to 23% of open liver resections,^8^, ^9^ while up to 15% of procedures result in irradical resections. These challenges are even more prominent in minimally invasive liver resections, where tactile feedback on organ’s structure is lacking. Thus, although laparoscopic liver resections may profoundly benefit patients undergoing abdominal surgery (i.e., short recovery time and lower morbidity),^10^ their integration into oncological surgical practice is still lagging due to shortcomings in intuitiveness (Fig. S1).

These shortcomings can be addressed by integrating image guidance into the surgical procedure (i.e., surgical navigation), where the surgical instruments’ positions is shown in relation to lesions and the critical surrounding anatomy. Moreover, it may help to improve the oncological outcome as well. Despite being the standard of care in orthopedic and neurosurgery,^11^ where the organ’s anatomy does not change substantially (e.g., rigid bone or brain encapsulated in the skull), surgical navigation systems for guidance on mobile and deformable organs such as the liver are still mainly applied within research setups.^12^, ^13^

Since its initial introduction in 1999,^14^ the generic principle of surgical navigation during liver surgery has remained largely unchanged: the intraoperative environment is registered to the pre‐operative plan, after which a sensor is used to track various surgical instruments.^15^, ^16^, ^17^ Despite many innovations introduced in the last 20 years, registration between the pre‐ and intra‐operative pose of the organ, as well as maintaining the registration accuracy throughout the resection, remain two of the navigation’s biggest challenges.^15^

Although various setups exist, all liver navigation systems can be roughly divided into two types: without (a) and with (b) involvement of tomographic intraoperative imaging (e.g., CT, tracked ultrasound or MRI). Due to fundamental differences in each of these setups, their approach to the registration challenge differs as well.

In the first group (i), surface‐based registration methods (e.g., based on an optical scan or landmark registration) are utilized to guarantee alignment between the surgical scene and the preoperative plan.^18^, ^19^, ^20^, ^21^, ^22^ This approach is largely applied in laparoscopic and robotic surgery, and shows the biggest potential for future integration into the augmented reality (AR) setup.^23^, ^24^, ^25^ However, because it does not allow for visualizing the liver’s underlying anatomy, accurate registration is only feasible prior to the start of the dissection (e.g., when the complete surface is intact) and its accuracy assessment is primarily based on evaluating liver surface alignment.^26^ Once the surgeon starts manipulating the organ, deformable biomechanical models are required to approximate the motion of both the underlying vasculature and the tumors. Although several groups are actively developing biomechanical liver models and have already illustrated very promising results,^22^, ^27^ the absence of intraoperative imaging in this group of navigation systems implies that the surgeon should fully rely on the accuracy of the guidance (e.g., projection of the underlying vasculature), resulting in slower widespread clinical acceptance of the approach.

The second method (ii) utilizes intraoperative (CB)CT, ultrasound or MRI imaging to provide a link between the preoperative plan and the liver’s intraoperative location, after which registration of the organ remains largely unchanged for the remainder of the resection. Unlike the first group (i), this approach allows incorporating the information about the liver’s actual intraoperative shape and location of its vasculature or tumors into the registration method.^16^, ^17^, ^28^, ^29^, ^30^, ^31^ As a result, the surgeon can visually asses the accuracy prior to the start of the guidance. Obviously, surgical manipulation of the liver will result in invalidation of the registration with the preoperative imaging. One of the most common approaches in tackling this challenge, is to perform multiple registrations throughout the resection, for example, using tracked ultrasound. Although this step enables surgical guidance at various timepoints during the surgery, it does not eliminate the problem’s cause, and results in prolongation of the surgery. Alternatively, active motion tracking (e.g., with optical or EM‐tracking) can be utilized to maintain the registration, yet is rarely used.

One of the major limitations of the currently available liver navigation techniques, is the lack of organ motion handling. Heizman et al.’s^32^ work suggests that although the total liver shape significantly changes throughout the surgery, the organ can be approximated as a locally rigid body (e.g., within one anatomical segment) with clinically acceptable accuracy.

Therefore, in this work, we develop and evaluate a new surgical navigation setup that incorporated CBCT‐based registration between the preoperative 3D model and the liver’s intraoperative pose (e.g., one timepoint match), while the target resection area is approximated as a rigid object and its motion is tracked via a single electromagnetic micro sensor.

## 2 MATERIALS AND METHODS

### 2.A Ethical approval and study design

This is an observational feasibility single‐center clinical study that was reviewed and approved by the Medical Ethics Committee (METC) of the Netherlands Cancer Institute Antoni van Leeuwenhoek hospital (NKI‐AvL) in May 2016. It is registered under the number NTR7019 in the Netherlands Trial Register and was open for patient inclusion between May 2016 and December 2018.

**Fig. 1:**
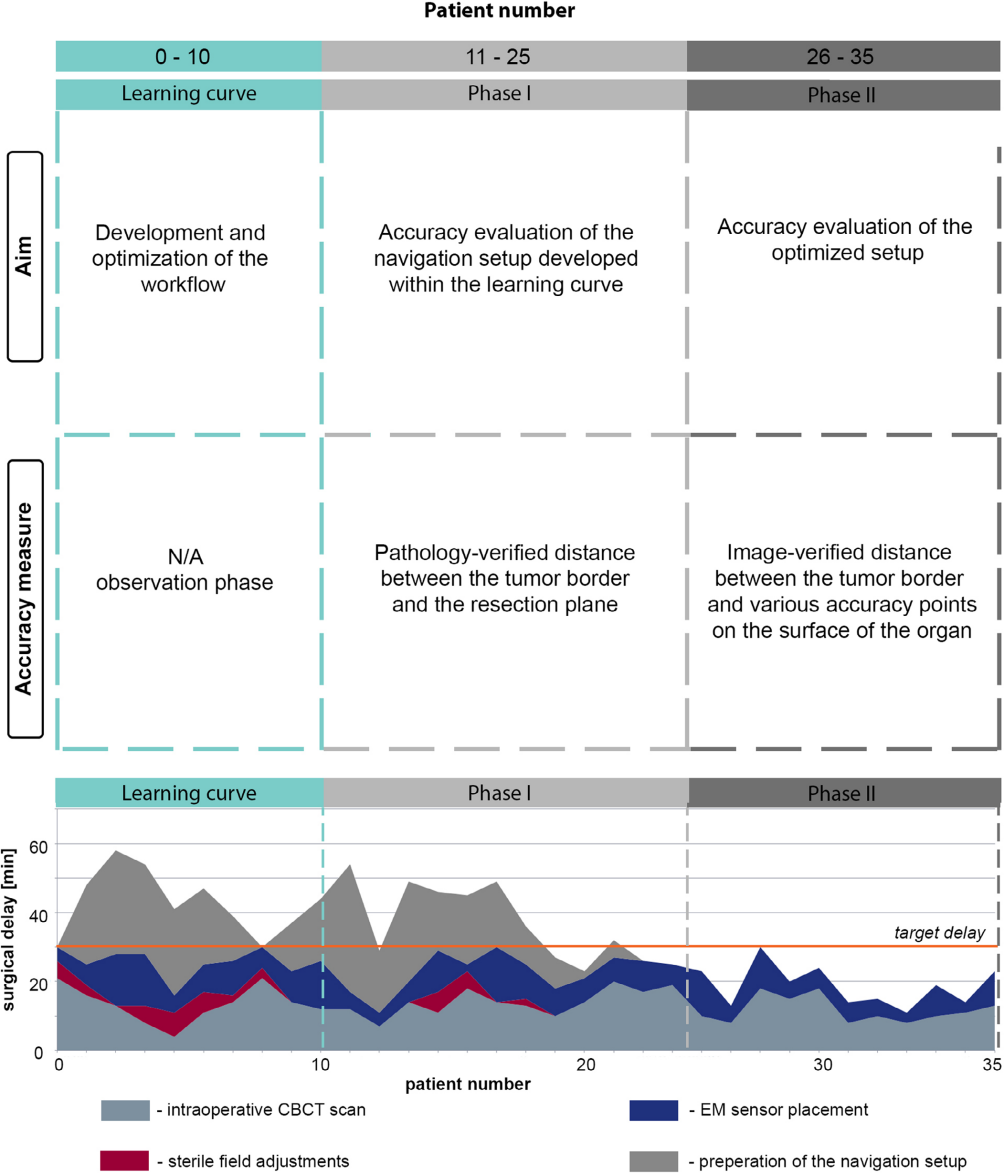
Schematic illustration of the study design (top), including definition of the aim and accuracy measurements which were used during each phase of the study. The bottom part of the figure corresponds to the graph of the total surgical delay caused by navigation‐related steps, and its distribution between various intraoperative tasks (color coding).

The study was divided into three clinical stages (Fig. 1). The first pilot stage is the initial "learning" curve, covering the first 10 patients of the study. It reflects the early stage of the study, with a still developing intraoperative workflow of the technique. The next two stages are the "navigation" stages, representing gradual methodological and technical advancement, and include 25 patients. Here, the navigation technique was used and quantitatively evaluated in the same way for all patients within the specified phase. Our goal was to reach a navigation accuracy of 5 mm. It was evaluated by assessing the accuracy of the shortest distance to the tumor, as indicated by the navigation (e.g., EM‐pointer to tumor edge distance). If achieved, this accuracy can help to guaranty recommended resection margins according to the national treatment guidelines of the CRLM patients.^33^, ^34^, ^35^, ^36^ More details are available in the Table S1.

### 2.B Technical setup, workflow and assumptions

#### 2.B.1 SurgNav navigation platform

The surgical navigation setup used in this work is an advanced version of the surgical navigation system introduced by Nijkamp et al.,^37^, ^38^, ^39^ which combines the Northern Digital Inc. Aurora EM‐tracking system^40^ with an in‐house developed navigation software (SurgNav). This navigation platform incorporates Embarcadeco Delphi XE2 for the guided user interface and C++ module libraries for image and data processing, while the EM‐tracking information is communicated via TRANFORM messages of the Plus Server (OpenIGTLink library).^41^ The SurgNav platform allows to feature up to five tomographic images (e.g., MRI or CT) with linked 3D models via DICOM‐RT structure format. Additionally, it can visualize up to eight electromagnetically tracked tools (e.g., surgical pointer, patient trackers) in the intraoperative guidance environment, with a 10Hz frame update rate (e.g., sensor and 3D model location). Further details on the setup and initial accuracy assessment of the SurgNav platform are available in Nijkamp et al. 2016.^37^

**Fig. 2:**
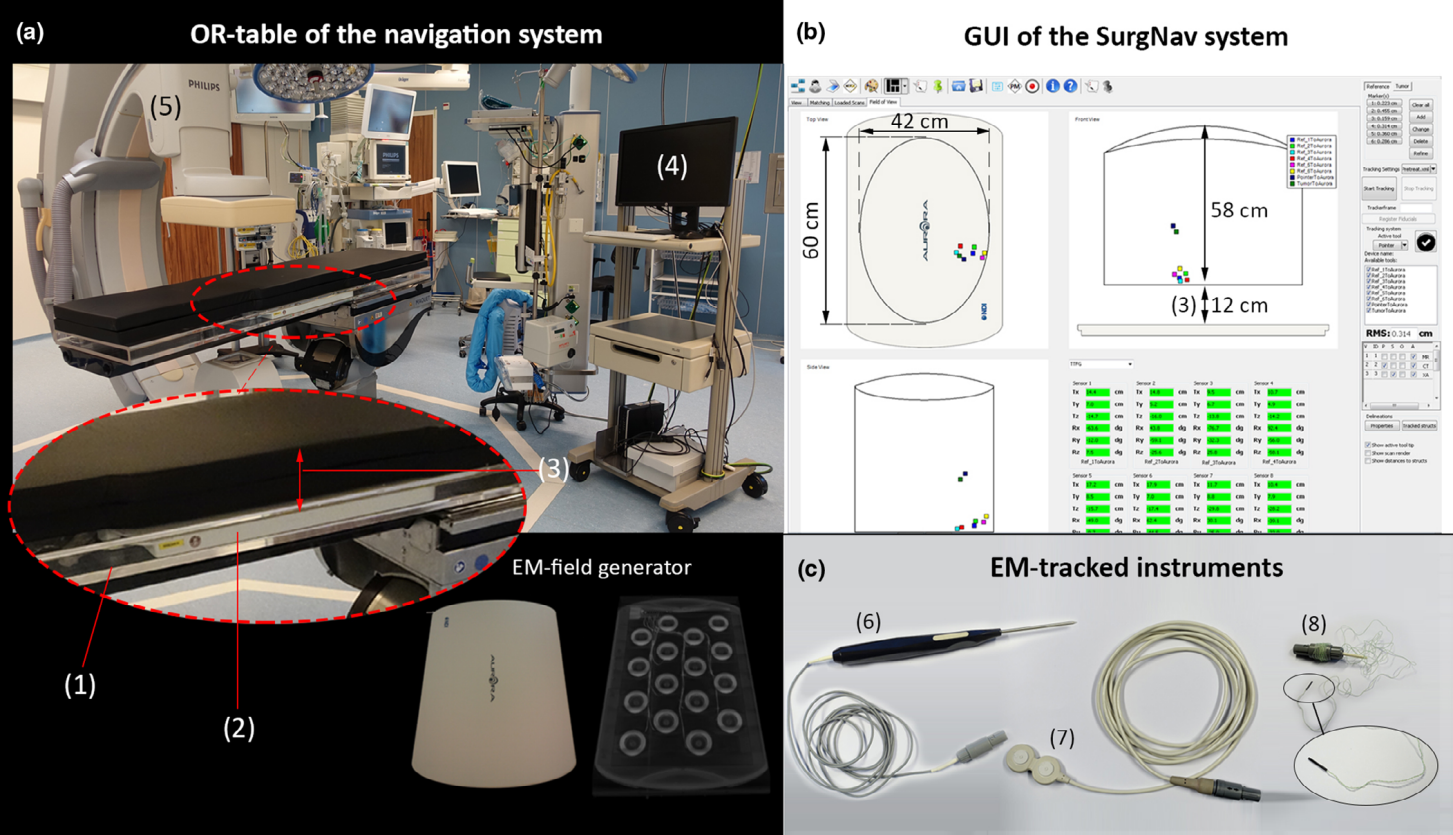
Components of the navigation setup. (a) OR table with a sliding compartment (1) for the EM‐field generator (2), back cushion for elevation of the patient to the level of EM‐tracking FOV (3), navigation trolley (4) and the CBCT scanner (5). (b) Guided user interface of the SurgNav navigation system, visualizing live position of EM‐tracked tools within the FOV of the EM‐field generator. (c) electromagnetically tracked tools of the setup, including EM‐pointer (6), external 5‐DoF patient‐trackers (7) and a micro‐6‐DoF EM‐sensor for tracking of the organ (8).

In contrast to the initial SurgNav software version introduced by Nijkamp et al.,^37^, ^38^, ^39^ the navigation system evaluated in this work was modified to enable intuitive guidance towards mobile targets (e.g., liver). To this end, the software was extended to allow for incorporation of multiple moving 3D models into the guidance interface (e.g., EM‐pointer and moving organs). Schematic illustration of the setup is provided in Fig. 2.

#### 2.B.2 Navigation setup of the study

**Fig. 3:**
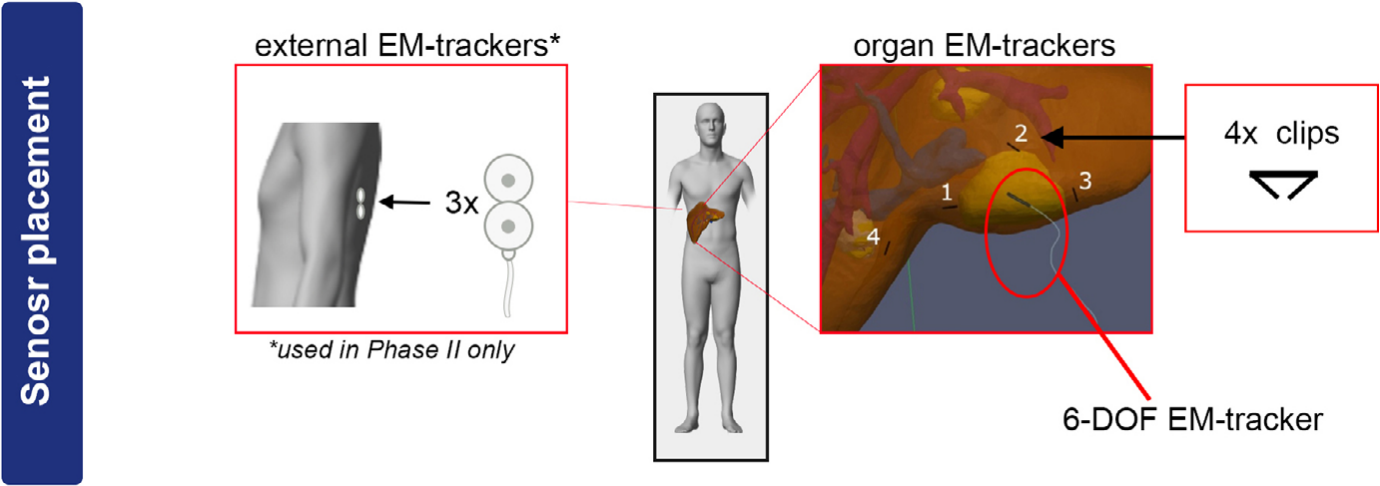
Schematic illustration of the sensor placement locations and ribs of the patient.

In the first two phases of the study (learning curve and phase I), the navigation system consisted of a tabletop field generator (Northern Digital Incorporated system, Canada), a six degrees of freedom (DoF) EM‐sensor for tracking of the target organ, an electromagnetically tracked surgical pointer, and a CT‐translucent operation table, containing a sliding compartment for positing and relocation of the EM‐field generator during the resection [Fig. 2(a)]. At a later stage of the study (phase II), three external electromagnetic patient trackers (PercuNav, Philips Healthcare, the Netherlands) were added to the setup [Figs. 2(c) and 3]. The complete list of hardware components of the navigation system can be found in Table I.

**Table I:** Hardware components of the navigation setup.

Item	Type	Number of components	Remarks	Study phase
Basic components				
EM field generator	NDI Tabletop generator	1	Active EM‐field generator	All
System control Unit	NDI SCU	1	Communication unit for the EM‐filed generator	All
Sensor interface unit	NDI SIU	2	Communication unit for EM‐tracked tools	All
EM‐tracked components				
Patient tracker	PercuNav external patient trackers	3	Three passive EM‐trackers, each contains two 5‐DoF sensors	Phase II
Organ tracker	Aurora micro 6‐DoF sensor	1	Passive micro 6‐DoF EM‐sensor	All
EM‐pointer	Aurora Blunt EM‐pointer	1	Sterilizable pointer with a single 6‐DoF sensor at the tip of the instrument	All

In order to allow for intraoperative tracking of the liver throughout the resection, we approximate the liver region near the EM‐sensor as a locally rigid body (e.g., roughly rigid within one to two anatomical segments).^32^ This entails that real‐time movement of the liver within close proximity from the EM‐sensor on the surface of the organ can be mimicked with a rigid affine transformation. The transformation corresponds to the live displacement of the 3D location of the EM‐sensor. Subsequently, a restricted area of approximately 5‐cm radius is defined as a “target navigation zone”, where we aim at achieving the navigation accuracy of 5 mm or better. Areas outside of the “target navigation zone” may still be used for visual indication of the underlying anatomy, yet will not be used for surgical guidance or calculation of the navigation accuracy.

### 2.C Imaging data

#### 2.C.1 Preoperative MRI and 3D model of the liver

All 3D models of the liver were created based on diagnostic Gd‐EOB‐DTPA‐enhanced (Primovist^®^ In Europe, Eovist^®^ in the USA, Bayer Healthcare, Germany) 3D FFE‐mDixon multiphase MRI‐scans acquired not earlier than 4 weeks prior to the surgery.^7^ Image acquisition contained five consecutive phases acquired during early enhancement of the contrast agent (i.e., pre‐contrast, early arterial, late arterial, portal venous, and intermediate phase), and one late‐phase showing hepatospecific filtration of the agent after around 20‐min post‐injection. Each phase is acquired with a 3D T1‐weighted FFE‐mDixon sequence, with a 12‐s single breath‐hold scan in expiration, and a reconstructed voxel size of 1.0 × 1.0 × 1.5 mm.

For each patient, a detailed patient‐specific 3D model of the liver, portal and hepatic veins, biliary tree and target tumors was extracted from magnetic resonance images (MRI) of the liver. In phase II of the study, a 3D model of the ribs was added to the model as well, using diagnostic CT.

Segmentation of the 3D models was performed by technical‐medical staff in our hospital (e.g., research assistant or a radiologic technologist), using an algorithm introduced in our previous work^7^ and incorporated into a custom module of 3D Slicer. The total model preparation time varied between 15 and 30 min, dependent on complexity of the case.

#### 2.C.2 Intraoperative steps: Preparation and CBCT

On the day of the surgery, each patient was positioned on a CT‐translucent OR table (MAGNUS, MAQUET Holding B.V., Germany) with an EM‐field generator build into the mattress of the OR table (Fig. 2). After this, for all patients within the initial “learning curve” and Phase I of the study, the surgery proceeded according to the standard clinical practice. In Phase II of the study, three additional external patient trackers, each containing two disc‐shaped 5‐DoF EM‐sensors, were placed on the back and posterior side of the ribs of the patients prior to the start of the surgery (Fig. 3). Next, the surgery proceeded according to the standard clinical procedure.

After laparotomy and mobilization of the liver, a single sterile 6‐DoF EM‐sensor and four surgical clips were attached to the surface of the liver in close proximity to the target tumor (Fig. 3). In order to provide a stable fixation of the EM‐sensor throughout the resection, it was inserted under the surface of the liver, yet never penetrated the edge of the tumor itself. This was done to minimize the chance of the “seeding” tumors in the remaining liver volume.^42^ The clips were required to enable real‐time tracking of the organ’s movement intraoperatively in phase I of the study (e.g., for fiducial registration), while in phase II, they were used to identify locations of future accuracy measurements on CBCT images. Subsequently, a sterile contrast‐enhanced CBCT‐scan without bolus triggering with a controlled breath‐hold, visualizing the sensor, clips and external patient trackers, was performed. EM‐field generator and metal instruments were kept outside of the FOV of the CBCT scanner, to minimize artifact in the reconstructed images. Details of the contrast injection protocol and timing of the CBCT scan are available in the SI.

#### 2.C.3 Intraoperative CBCT data and truncated projection reconstructions

**Fig. 4:**
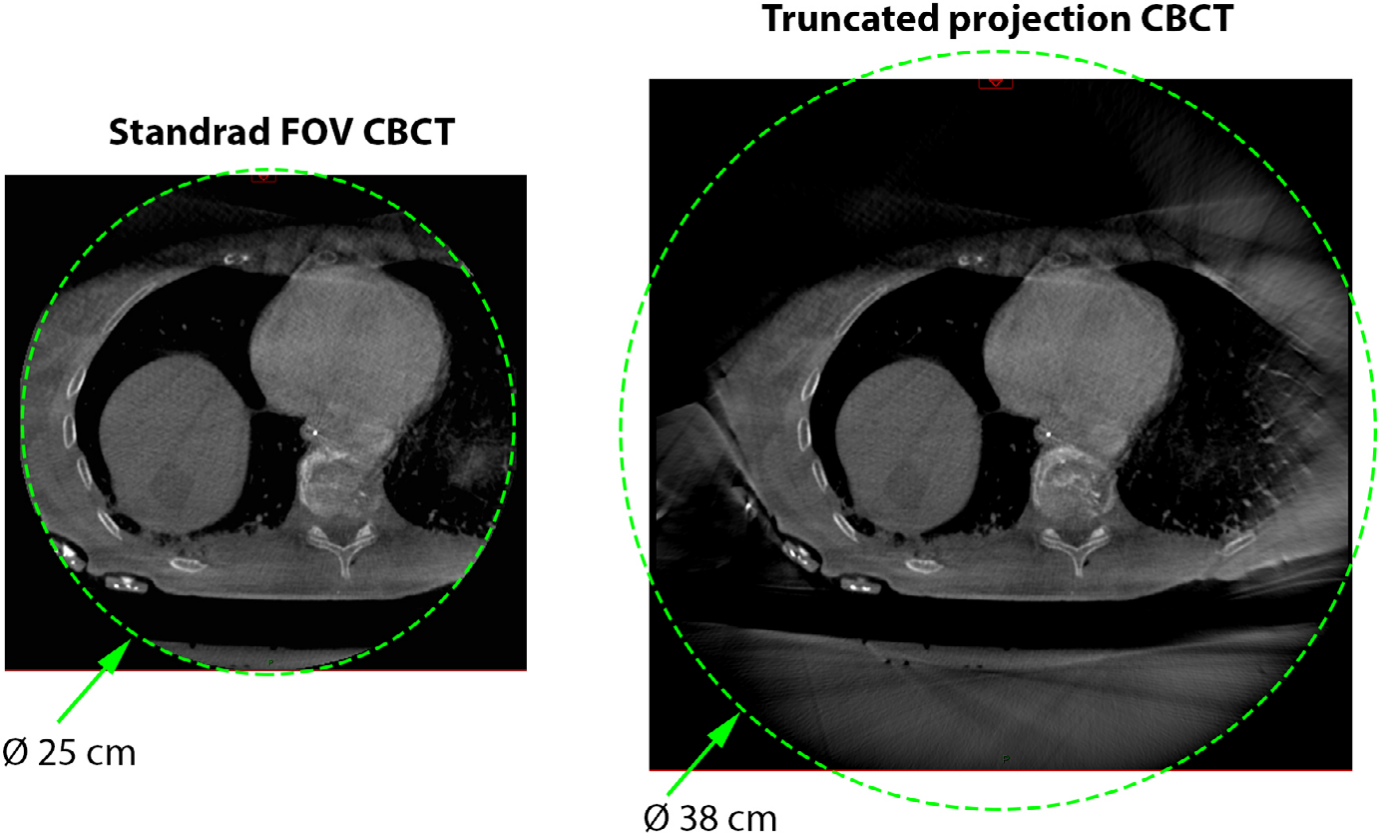
Comparison of the standard Ø25 cm FOV (left) and truncated projection FOV (right) reconstructions.

All intraoperative CBCT data were acquired with an Allura FD‐20 monoplane C‐arm system (Philips Healthcare B.V., Best, The Netherlands), integrated in the hybrid OR of the NKI‐AvL. The data were acquired with the XperCT dual‐phase roll scan protocol and reconstructed on a 0.66 × 0.66 × 0.66 mm voxel grid. Two types of CBCT reconstructions were used in this work: standard clinical field‐of‐view (FOV Ø 25 cm, length = 20 cm) and truncated projection FOV reconstruction (FOV = 35 × 35 × 20 cm), used within phase I and phase II of the study, respectively. Truncated FOV reconstruction corresponds to a non‐cropped image volume, extending beyond fully sampled central Ø 25 cm part of the image up to the edge of a 35 × 35 × 20 cm cube (Fig. 4). A capability of the truncated FOV reconstruction was enabled within the research collaboration with Philips Healthcare, as specified in the acknowledgements.

**Fig. 5:**
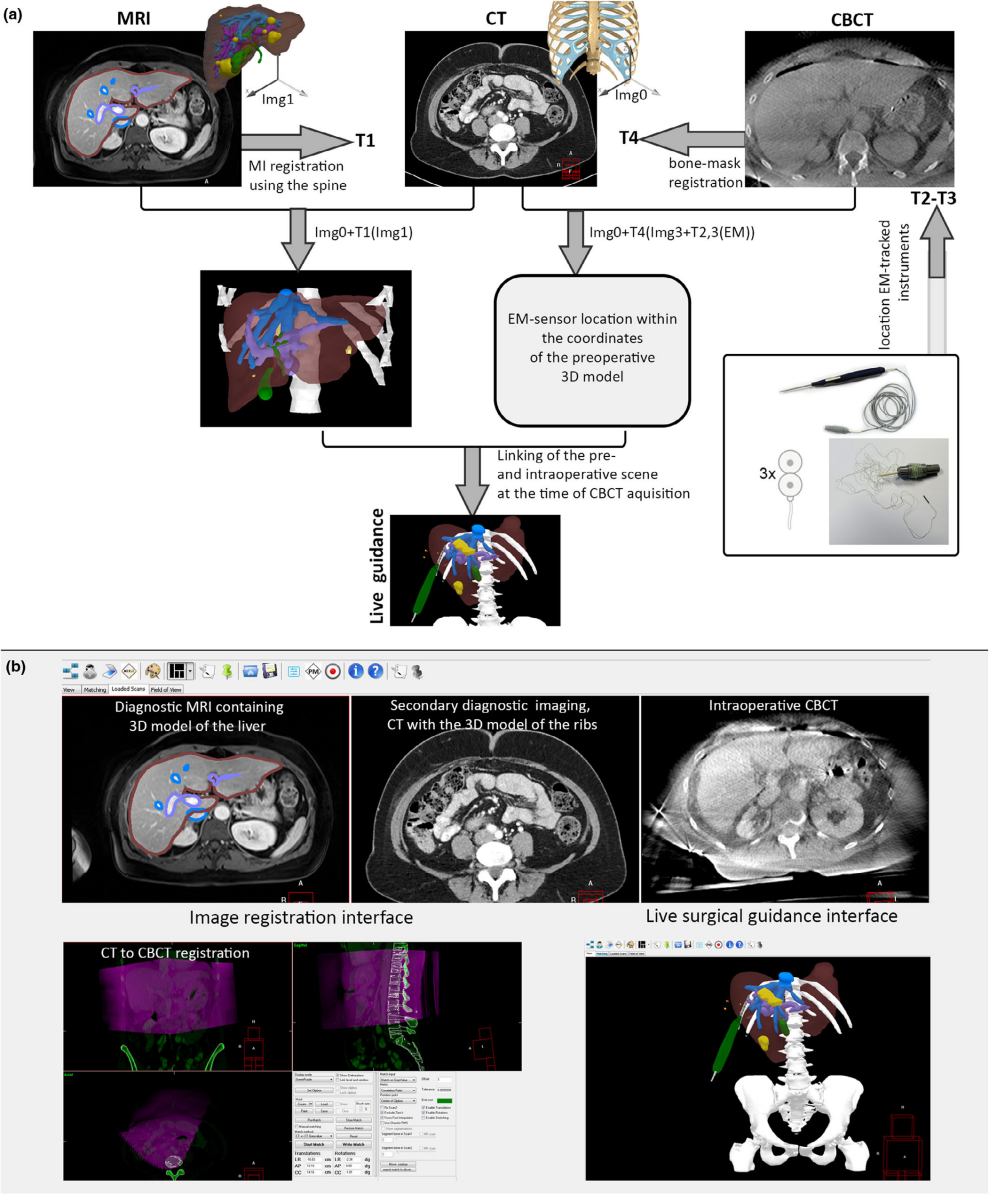
(a) Schematic illustration of the registration pipeline, used to bring the pre‐operative model of patient anatomy, intra‐operative CBCT and EM‐tracking to the shared coordinates system of the surgical navigation software (SurgNav). (b) Illustration of three GUI modules of the SurgNav software, involved in the registration pipeline (b — top, bottom left) and surgical guidance during the surgery (b, bottom right).

Under sampled areas of the CBCT image, extending beyond clinical Ø 25‐cm image, were used for localization of external patient trackers only. The trackers were added to the setup in Phase II of the study. Possible geometrical distortions of the truncated projection CBCT reconstruction were evaluated prior to the first clinical use of the method in the study. This was done by measuring the absolute difference between automatically detected centers of external patient trackers (Fig. 5) in fully sampled and truncated projection reconstructions, using patient tracker detection method incorporated in the SurgNav software.^38^, ^39^ The total displacement between sensor’s location calculated for two reconstruction types was used for qualitative assessment of the distortion. The effect was found to be lesser than the smallest pixel size of the CBCT data (mean displacement of 0.2 mm, see SI and Table S3), thus was considered to be negligible.

#### 2.C.4 Registration of the 3D model to the CBCT: Learning curve and Phase I

Directly after the end of the CBCT acquisition, the data were reconstructed (standard CBCT reconstruction) and transferred to the navigation station. Subsequently, the CBCT was manually rigidly registered to the preoperative MRI scan containing the 3D model, taking only the area around the target tumor into an account (e.g., restricted ROI registration). After this, a point‐based registration using four fiducials between the CBCT and the EM‐tracking system was achieved by matching the center of each surgical clip visible in the CBCT scans (manual localization by researcher), with the locations of electromagnetically tracked surgical pointer placed on the corresponding surgical clips on the surgeon, respectively.

#### 2.C.5 Registration of the 3D model to the CBCT: Phase II

Directly after the end of the CBCT acquisition, the C‐arm was moved outside of the scan FOV, while keeping mechanical ventilator controlled breath‐hold of the patient, and the 3D location of EM‐sensor on the liver surface of the liver and three external patient trackers was saved in the system. This information was later used to determine the exact location of the liver and the ribs during acquisition of the CBCT image.

After reconstruction and transfer of the truncated projection CBCT image, mutual information (MI) based bone‐to‐bone CT‐to‐CBCT registration was performed (Transformation 4, Fig. 5). It consisted of rough automated masking of the images within expected grey value range of the bones. Subsequently, the registration was initialized by rigidly alignment between COM of two bone masks, and MI‐based registration was performed, using masked areas of the image only. This transformation allowed registration of the rib’s 3D model within the CBCT scan. Next, location of the center of each disk‐shaped 5‐DoF sensor within the external patient trackers (two sensors per patient tracker, six 5‐DoF sensors in total) was automatically detected in the CBCT and registered to the real‐time location of the sensors, by minimizing the RMS errors of the six points (T2–T3, Fig. 5).^38^, ^39^ This step enables real‐time tracking of the ribs and patient’s body. Subsequently, MRI volume containing the 3D model of the liver was manually rigidly registered to the intraoperative contrast‐enhanced CBCT, based on vascular tree anatomy around the target lesion (T1, Fig. 5). Here, locally rigid anatomy within the area of resection was assumed. The real‐time tracking of the target tumor and the surrounding anatomy was enabled by linking orientation of the 3D model in the co‐registered MRI scan to the location of the 6‐DoF EM‐sensor of the liver, as saved at the end of the intraoperative CBCT acquisition. Schematic illustration of the registration pipeline is provided in Fig. 5(a).

#### 2.C.6 Accuracy measurements

Directly after the completion of the MRI‐to‐CBCT registration and start of the intraoperative tracking of the organ, accuracy of the navigation system was qualitatively checked by a surgeon. This was done by pointing at various anatomical landmarks (e.g., vessel bifurcations or liver surface) with an EM‐tracked surgical pointer, and comparing this location with the one indicated by the navigation system on a computer screen (Fig. 4, Video S2). These qualitative accuracy controls were performed throughout the resection.

**Fig. 6:**
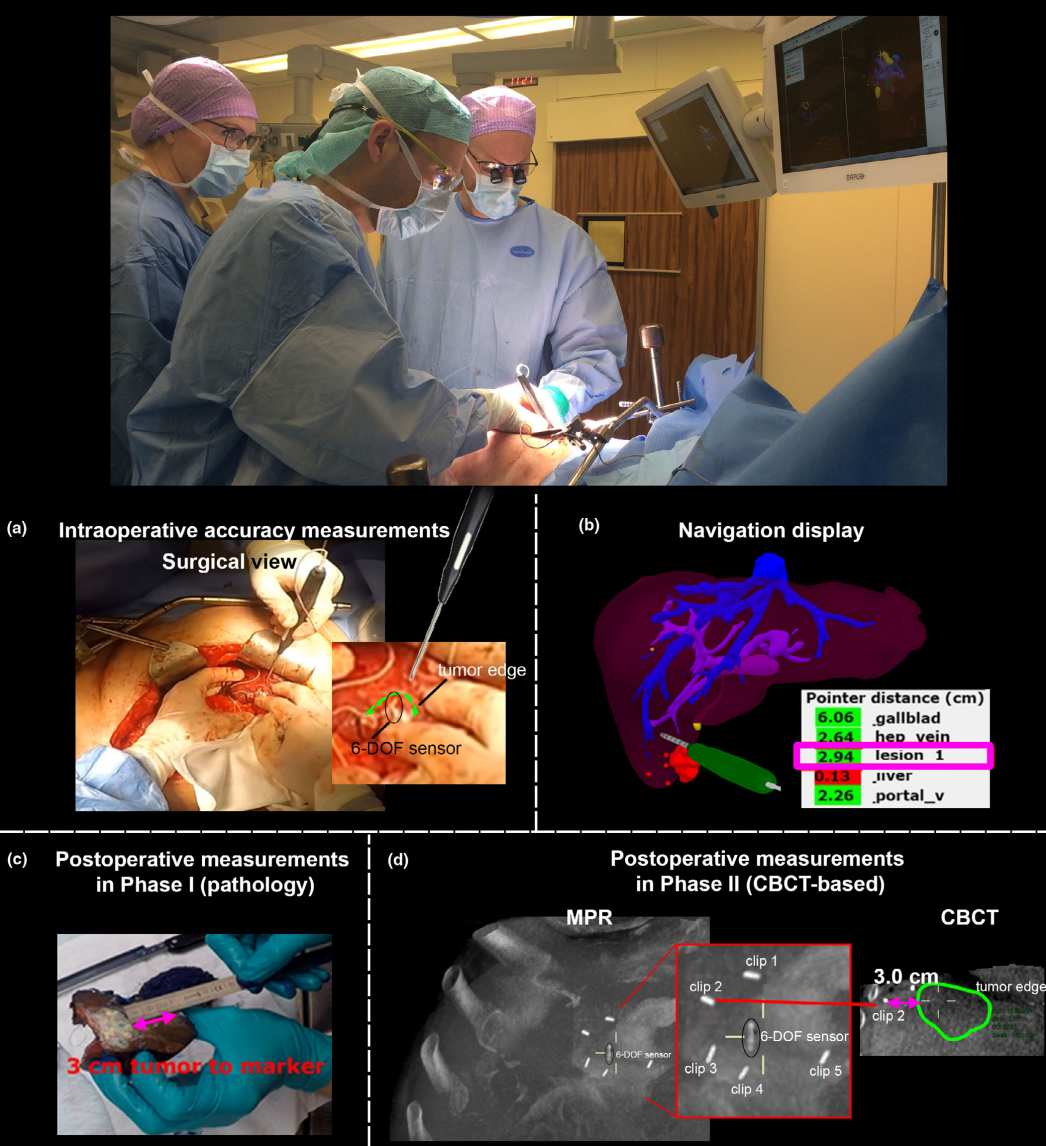
(a, b) Location of EM‐pointer during intraoperative accuracy measurement and corresponding navigation software display. (c) Postoperative accuracy measurements on resected ex vivo pathology specimen (Phase I). (d) Postoperative accuracy measurements on CBCT images (Phase II).

In addition to repetitive visual checks, the accuracy of the system was quantitively assessed in two different ways for Phase I and Phase II of the study (Fig. 6). In both Phase I and Phase II, the surgeon placed a few surgical markers (sutures in phase I and clips in phase II) within the resection plane of the liver to mark the location of the measurement (Figs. 3 and 6). After placement of the markers, the navigation pointer was used to record the position of the markers and calculate the shortest distance between the marker and the tumor lesion according to the navigation system [Figs. 6(a)–6(b)].

In phase I of the study, this shortest distance was compared with the corresponding distance postoperatively measured on the specimen during pathological examination [Fig. 6(c)]. In phase II of the study, this shortest distance was compared with the corresponding distance measured on CBCT images [Fig. 6(d)]. To measure these distances on the CBCT, three expert observers independently measured the distance between the center of each clip and the closest visible edge of the target tumor. The absolute difference between the navigation‐based distance and the average distance measured postoperatively on the CBCT by the observers was reported as the accuracy measure.

## 3 RESULTS

### 3.A Patient inclusion

A total of 35 patients were included in the study between September 2016 and June 2018. Selected patients were scheduled for open liver surgery, had superficial liver lesion(s) with a diameter of at least 2 cm, had recent CT or MR scans, did not have a pacemaker or metal implant, were not allergic to iodine contrast, and their estimated glomerular filtration level (eGFR) was above 60 ml/min. A complete list of patient inclusion criteria and patient division per study phase is provided in Table S1 (SI).

### 3.B Learning curve

Within ten navigation procedures performed in the initial learning curve of the study, a basic clinical workflow was developed (Video S1). This included but was not limited to the contrast‐enhanced CBCT protocol (incl. timing, volume and automatically controlled breath hold), logistics of the sterile CBCT scan during the resection, and attachment method for the EM‐sensor on the surface of the liver. Several sensor attachment techniques were evaluated, including use of adhesive surgical glues [Fig. 6(a)], suturing of the sensor to the surface of the organ, and insertion of the sensor into the liver parenchyma [Fig. 6(d)]. The latter option provided the best stability of the fixation throughout the procedure, in particular with respect to radial rotation of the sensor, and therefore was used in the following phases of the study.

Within the learning curve, only four procedures were considered as complete, meaning that they included an intraoperative CBCT acquisition, a surgical guidance session and the proof of concept pathology‐based accuracy measurements.

The main challenge was related to the poor soft tissue contrast of the CBCT, caused by sub‐optimal timing of the contrast, yet was resolved by the end of the learning curve phase. A more detailed description of the developed clinical workflow is provided in Table S2, while Fig. 1 illustrates the average surgical overhead time for this stage of the study.

### 3.C Phase I

#### 3.C.1 Quantitative results

After development of the initial clinical workflow, a total of 15 navigated procedures were performed within phase I of the study. One procedure was counted as a technical failure, due to an accidental damage of the EM‐sensor on the surface of the liver by a diathermia during the resection. Therefore, this case did not offer any further data. The surgical overhead time of the remaining 14 procedures was 32 min (Fig. 1). This includes placement of the EM‐sensor and surgical clips on the surface of the liver (8.5 min), sterile intraoperative contrast‐enhanced CBCT scan (14 min), registration of the 3D model with a real‐time situation and all navigation‐related measurements (9 min). Three more cases did not result in complete accuracy measurements: one due to the logistics complication at the pathology department, two due to accidental damage of the organ tracker during the accuracy measurements. Therefore, although surgical guidance was performed in 15 cases, only 11 of them resulted in complete procedures with pathology‐verified accuracy measurements.

The navigation setup within Phase I resulted in visually accurate and intuitive real‐time visualization of the liver anatomy and tumors’ location (Video S2), confirmed by intraoperative checks on visible anatomical landmarks. Nevertheless, based on 43 quantitative accuracy measurement verified by the pathology (i.e., 3–4 locations per patient), the average accuracy was only 11.8 ± 11.2 mm and had low correlation with the gold standard (Table II).

**Table II:** Quantitative assortment of the navigation setup.

Criteria	Phase I	Phase II
Number of procedures (surgeries)	15	10
Number of complete procedures	11 (73%)	9 (90%)
Number of accuracy measurements	43	40
Accuracy [mm]	11.8 ± 11.2^a^	4.0 ± 3.0
R‐score and P‐value of the accuracy^b^	0.18 (p = 0.24)	0.94 (p < 0.00001)
Average depth of the tumor and its IQR^c^ [mm]	21 (2; 29)	14 (2; 25)
Average tumor diameter its IQR [mm]	33 (19; 44)	42 (19; 56)
Number of procedures performed, based on the liver segment containing the target tumor	S2 (7%)	S2 (0%)
	S3 (20%)	S3 (20%)
	S4 (13%)	S4 (10%)
	S5 (7%)	S5 (10%)
	S6 (20%)	S6 (20%)
	S7 (27%)	S7 (20%)
	S8 (7%)	S8 (20%)
Total surgical delay [min]	32	20
CBCT scan, including sterile field adjustments [min]	14	12.5
Sensor placement [min]	8.5	6.5
Intraoperative interactions [min]	9	—
EM — to CBCT coordinates registration	Fiducial registration	EM‐based organ tracking during CBCT scan
Accuracy measurement	Tumor to resection plane distance^d^	Tumor to the surface of the liver^e^
Accuracy measure	Guidance accuracy within the resection plane	Generic guidance accuracy

^a^ From the surface of the organ to the edge of the tumor.

^b^ Significance level of 0.05.

^c^ IQR – inter quantile range (Q1, Q3).

^d^ Distance between tumor edge and surgical suture, place on the surface of the organ during accuracy measurement.

^e^ Distance between tumor edge and center of the surgical clip, placed on the surface of the organ prior to CBCT scan.

Moreover, there was a high variation between quantitative accuracy measurements within one patient (1.5–15 mm variation within one patient), even for measurement locations within close proximity to each other. Lack of consensus between qualitative and quantitative accuracy controls, as well as high variation of the results raised questions about reliability of the method for accuracy measurements used within Phase I.

#### 3.C.2 Limitations of accuracy measurements

Initially, pathology‐based accuracy measurement were selected as a “gold” standard due to the wide acceptance of this method within navigation‐ and image guidance‐related studies.^43^ However, this method is ultimately affected by the inter‐observer variation of pathologists and tissue deformation of *ex vivo* samples. In order to estimate these variations, the closest distance between the clips and the edge of the tumor was calculated using the pathology method and the CBCT method (as described in the methods) for patient 21 to 25. To increase robustness of the results, measurement of each accuracy point was repeated three times (average interobserver variation of 2,5 mm). Based on 12 comparative measurements (e.g., three points per patient), pathology‐based measurements deviated by 20 ± 14 mm from the image‐based measurements (Tables S4 and S5) and had up to 5 mm spread within three repeated measurements of the same point (~10 % variation).

The main reason for such a variation could have been sub‐optimal slicing of the liver specimen in the direction of the target tumor (e.g., not a cross‐section with the shortest distance between the edge and the tumor). As a result, it was decided to switch to a different method of quantitative accuracy evaluation in Phase II of the study.

#### 3.C.3 Feedback of the surgeon: Intuitiveness of the setup and total surgical delay

In addition to qualitative and quantitative accuracy measurements throughout the resection, the navigation setup was regularly evaluated based on surgeons’ feedback on intuitiveness of the intraoperative guidance (Videos S2 and S3), and general satisfaction with the total surgical delay.

Within Phase I of the study, the liver model was visualized as a static 3D object on the screen of the navigation computer, while all movements of the EM‐tracked surgical pointer were visualized in real‐time (Videos S1 and S2). This setup was tested during the learning curve and was selected as a good candidate for the initial navigation interface. However, based on uniform feedback of three liver surgeons involved in the first phase, addition of a realistic movement of the liver model was requested prior to the start of the second phase. This was achieved by adding three external patient trackers, which were linked to the position of the ribs (Fig. 3). Subsequently, all movements of the liver with respect to the ribs were approximated based on the real‐time location of the 6‐DoF EM‐sensor on the surface of the liver (<5 cm from the tumor). This change also required the use of an extended FOV CBCT reconstruction with partially truncated projection data. Because co‐registration between preoperative 3D model and an intraoperative location of the liver necessitates visibility of all external patient trackers on the ribs and the liver itself on the intraoperative CBCT (see methods), it is not possible to fit this anatomical region within the standard clinical CBCT reconstructions of Ø 25 cm.

Second concern of the surgeons was related to the total overhead time caused by the navigation technique. In Fig. 1 a task‐wise division of the total overhead time per patient is provided. Main contributing factors of the delay were intraoperative tasks of the surgeons (i.e., setup of the navigation, Fig. 1), including point‐based registration between the preoperative 3D‐model, CBCT scan and the real‐time tracking, as well as qualitative controls of the accuracy. However, as one can see from Fig. 1, the speed of the point‐based registration and the number of qualitative checks decrease drastically throughout the duration of the first phase. We attributed this drop to the still present learning curve of the surgeons (i.e., for point‐based registration) and increasing trust in the navigation system (i.e., less qualitative controls). Nevertheless, in order to further shorten the total overhead time and increase accuracy in Phase II of the study, point‐based registration between the pre‐ and intra‐operative situation was replaced by an automatic detection of the organ’s location with respect to the ribs of the patient, as described in Methods.

Time required for sterile intraoperative CBCT acquisition was the second greatest contributing factor to the total overhead time, yet did not change significantly after the initial learning curve.

#### 3.C.4 Phase II: Results, limitations and clinical applications of the techniques

After completion of Phase I and implementation of all technical adjustments to the navigation setup, a total of 10 navigated procedures with image‐verified accuracy measurements were performed within Phase II of the study. Main changes in the setup were related to registration and accuracy verification methods, as described in the previous section. Results of this phase illustrated significant reduction of the total surgical delay (Table II) and accuracy improvement of the system, when compared to Phase I. Based on 40 accuracy measurements (9 patients) verified by intraoperative CBCT, the average accuracy of the system was 4.0 ± 3.0 mm, representing clinically acceptable accuracy range with statistically significant results (R = 0.94, *P* < 0.00001). Additionally, the system was successfully able to handle organ manipulations (Videos S3 and S4). All ten procedures resulted in an accurate guidance throughout the resection. However, only nine out of them had quantitative measurements. One incomplete procedure was caused by a sub‐optimal position of the patient on the OR table, which prevented imaging surgical clips on the surface of the organ (e.g., accuracy points), therefore obstructing following image‐based quantitative accuracy verifications. The total surgical delay of navigated procedures decreased to 20 min (Table II), representing a decrease of 12 min, when compared to Phase I. Additionally, all liver surgeons included in the study were satisfied with the addition of realistic liver movement with respect to the ribs of the patient, what helped to improve intuitiveness of the guidance (Video S3).

## 4 DISCUSSION

This work focuses on the clinical evaluation of surgical guidance during open liver resections by means of simplified organ approximation as a semi‐rigid body and EM‐based tracking of the motion of the liver. Our assumption was that already a semi‐rigid organ model will result in clinically acceptable accuracy of the navigation (e.g., <5 mm), if the location of the target area is continuously updated throughout the resection. In a way, the proposed setup represents a simplified mix between the two types of liver navigation (i, ii) previously discussed, where temporal changes in the semi‐rigid model of the organ are measured via an electromagnetic tracking (i.e., simplified biomechanical model). The setup was extensively evaluated and updated within a clinical study including 35 patients, and resulted in an accuracy of 4.0 ± 3.0 mm in the last ten patients, verified by intraoperative CBCT. Additionally, the system was successfully able to handle organ manipulations (Videos S3 and S4).

At the beginning of the study, an assumption about the locally rigid anatomy of the liver was made. This assumption was based primarily on previous work of Heizman et al.,^32^ which investigated the deformation between the pre‐ and post‐laparotomy shape of the liver on CT images. Such a system design choice implied that the intraoperative imaging data should be acquired with minor or no deformation of the area of interest with respect to the reference diagnostic scan containing the 3D model. Additionally, the study placed no restriction on location of the target tumor, ultimately requiring imaging access to the complete volume of the liver, including segments VII and VIII (e.g., wide FOV imaging like CBCT).

Despite the simplicity of the semi‐rigid organ approximation, our results suggest that it allows for accurate guidance throughout the resection, when the location of the organ is being tracked. This statement partially contradicts recent work from Prevost et al.^18^ (AR‐based laparoscopic setup) stating that deformable registration between the resection plan and the intraoperative image is essential in reaching clinically acceptable accuracy. We would like to stress that the navigation setup suggested in this work contains several fundamental design differences, compared to Prevost et al., which justify our conclusions. First, our study restricted the navigation area of the liver to a zone of R = 5 cm around the organ tracker; this restriction complies with constraints on the semi‐rigid model of the organ. Secondly, our setup measures temporospatial motion of the target zone by means of a 6‐DoF micro EM‐sensor, allowing to compensate for surgical‐manipulation and breathing‐related movement of the organ. High accuracy of our navigation setup indicates that the active tracking of an organ’s motion during resection has a major effect on the accuracy of the guidance. Implementation of deformable image registration is definitely expected to improve this accuracy,^44^ although only as a secondary measure.

The majority of surgical navigation setups for liver surgery found in the literature involve intraoperative ultrasound imaging.^15^ This is primarily because this imaging modality is already a part of standard clinical workflow, requiring minimal changes to the surgical setup. Additionally, it is an affordable and non‐ionizing type of imaging. Despite these advantages, only a few US‐based setups were able to reach guidance accuracy well below 1 cm,^13^, ^18^, ^28^ and none of them was actively able to track the motion of the liver. As we have illustrated in this work, the tracking of an organ’s motion has a profound effect on the total accuracy of the navigation setup. Continuous US data can be used for image‐based tracking of the target tumor and surgical instrument during open resections as well. However, actual clinical implementation of such a setup will face several challenges. First, intraoperative US is primarily a 2D imaging modality due to physical restraint on the size of transducers suitable for surgical use. Therefore, utilization of US‐based organ tracking will require live and accurate 2D‐to‐3D image registration.^45^, ^46^ Several MICCAI conferences have tried to address this challenge within CLUST workshops (https://clust.ethz.ch).^47^, ^48^, ^49^ Comparative results for CLUST have illustrated that although 2D US‐based tracking of the liver is feasible within 3 to 17 mm of localization error range for percutaneous applications, only 3D‐based methods result in clinically acceptable accuracy (e.g., <5 mm).^50^ These errors are expected to be larger during liver resections due to a higher range of motion. Secondly, image‐based organ tracking with US requires stable longitudinal contact between the transducer and the surface of the liver, typically imposing a transducer‐holding burden on a surgeon. Robotic arm‐assisted sonography can help to automate this step,^51^ yet it is infrequently used in open surgical setups (i.e., the challenge of robust pressure feedback). In this work, the conceptual principle of a single time‐point registration and sensor‐based tracking of the liver was evaluated and showed a clinically acceptable accuracy. The principle is translatable to other imaging modalities, including ultrasound. Therefore, although US‐based guidance does not yet allow for image‐based tracking of mobile targets, this limitation may be eliminated through sensor‐based tracking of the motion.

Evaluation of the accuracy of navigation is a challenging task, particularly due to the lack of standard reference during surgery. Surface‐based assessment of Fiducial Registration Error (FRE) or evaluation of Tumor Registration Error (TRE) using intraoperative ultrasound are two of the most commonly used methods. In this work, the accuracy of the shortest distance to the tumor, as projected by the SurgNav, was assessed using pathology (Phase I) and CBCT (Phase II). Pathology is a well‐accepted standard for assessment of resection margins; therefore, it was selected as the initial accuracy reference in the study. However, we illustrated that even these expert measurements are affected by intra‐observer variability (e.g., 10% variation during repetitive measurement of the same point). This effect is expected to be even stronger for FRE‐based accuracy assessment, due to interobserver variability of fiducial identification by surgeons, and it therefore should be taken into an account during design of the study. Additionally, deformation of *ex vivo* specimens was found to be a significant contributing factor to the total variability of accuracy measures. Our results suggest that the selection of the method for assessing accuracy strongly influences reported navigation accuracy of the setup due to various biases that are embedded in each of the methods for measuring accuracy. Meaningful comparison of different navigation setups necessitates introduction of standardized accuracy assessment techniques, as well as quantitative assessment of the biases incorporated in these methods.

The main target group that can benefit from surgical navigation are patients who require complex resections with centrally located tumors, generic liver resections with the involvement of major biliary tree or hepatic vasculature branches, and laparoscopic resections. In the first case, target tumors can be located up to ~6 cm from the surface of the organ, dependent on the total size of the tumor. In phase II of the study, the average distance between the organ tracker (EM sensor) and the edge of the target tumor was 3 cm (measured on intraoperative CBCT). Therefore, it should still be feasible to provide an accurate guidance for centrally located lesions. However, it might require placement of the sensor on the posterior side of the organ or inside liver parenchyma, to minimize the sensor‐to‐tumor distance. In the second case — resections with involvement of central vasculature or biliary branches — accurate guidance may increase the speed of parenchymal resections for a broad range of clinical applications, also beyond oncological applications used in this work. Moreover, it may help to reduce the number of postoperative complications related to unintended damage of the biliary tree (i.e., due to an unknown anatomical variation).^52^ With respect to laparoscopic navigated surgery, the navigation setup suggested in this work has a good potential for further clinical development. Electromagnetic tracking of instruments (e.g., laparoscopic US transducer) is rarely used during laparoscopic procedures due to the challenging incorporation of wired EM‐sensors into the resections field (e.g., via the access port). However, recent work by Eppenga et al.^53^, ^54^, ^55^ has illustrated that accurate EM‐tracking of mobile targets is feasible by means of two 5‐DoF wireless EM‐transponders (Calypso, Varian Medical Systems Inc). The EM‐transponders could be implanted preoperatively in a percutaneous approach at the radiology department under US or CT guidance. This step could further improve the intraoperative workflow. The EM‐tracking field of view of the Calypso system is not yet able to cover the complete resection field; however, this limitation is expected to be addressed in the near future.^54^

Despite high the accuracy of our navigation setup (Phase II), widespread clinical applicability of the approach for open liver resections is challenging, because it requires sterile intraoperative CBCT imaging. Intraoperative CBCT imaging was also the main contributing factor of the total surgical delay throughout the study, which plateaued around 12.5 min after the initial learning curve. This ultimately limits usage of the navigation setup to hybrid operating rooms containing mobile CT scanners (or 3D C‐arms), and restricts application of the navigation to one target area per resection (e.g., within close proximity of EM‐sensor imaged within the CT). Additionally, although CBCT‐based guidance on various anatomical regions is technically possible, it will require re‐attachment of the 6‐DoF EM‐sensor within close proximity of the second target area, and acquisition of the new CBCT scan, ultimately causing relatively high surgical delay and an extra radiation dose for the patient. These challenges can be partially eliminated by replacing intraoperative CBCT with tracked ultrasound imaging, even if the rest of the navigation setup remains unchanged. Such a transition is currently under investigated in our group.

## 5 CONCLUSIONS

The electromagnetic‐navigation system developed in this work allows for accurate localization of liver lesions and critical anatomy surrounding the resection area during manipulation of the organ, by means of EM‐based tracking of the motion. Our results illustrate that liver tissues can be approximated as a locally rigid body within a restricted anatomical area. The navigation approach introduced in this work can be adapted to navigation on other mobile and deformable organs and therefore may benefit various clinical applications.

## ETHICS STATEMENT

The study was performed with an approval of the Medical Ethics Committee (METC) of the Netherlands Cancer Institute Antoni van Leeuwenhoek hospital (NKI‐AvL) in May 2016. It is registered under the number NTR7019 in the Netherlands Trial Register and was open for patient inclusion between May 2016 and December 2018. Each patient signed an informed consent prior to inclusion into the study.

## CONFLICT OF INTEREST

The authors declare no competing interests.

## DATA STATEMENT

The data and software used for the current study are available from the corresponding author (OVI) upon reasonable request and through collaborative investigations.

## Supporting information


**Fig. S1**. Division of all liver procedures for malignancies, performed in the Netherlands between 2014 and 2016, between minor (<2 segments) and major (>2 segments) resection types.

**Table S1**. Patient inclusion criteria.

**Table S2**. Summary of the main navigation‐related steps of the study, as was defined at the end of the learning curve phase.

**Table S3**. Comparison of pathology‐ and CBCT‐based accuracy measurements with respect to the output of our navigation system. Measurements were performed on the same locations.

**Table S4**. CBCT‐based accuracy measurement of three independent observers.

**Table S5**. CBCT‐based accuracy measurement of three independent observers.

**Video S1**. Navigation setup within the learning curve.

**Video S2**. Navigation setup ‐ Phase I.

**Video S3**. Navigation setup ‐ Phase II (organ motion).

**Video S4**. Navigation setup ‐ Phase II.

**Data S1**. Study design and sample size calculation.
