# Supplementary figures and images for: CBCT‐based navigation system for open liver surgery: Accurate guidance toward mobile and deformable targets with a semi‐rigid organ approximation and electromagnetic tracking of the liver
Source: Med Phys. 2021 Apr 1;48(5):2145–59. doi: 10.1002/mp.14825 (PMC8251891; doi:10.1002/mp.14825)

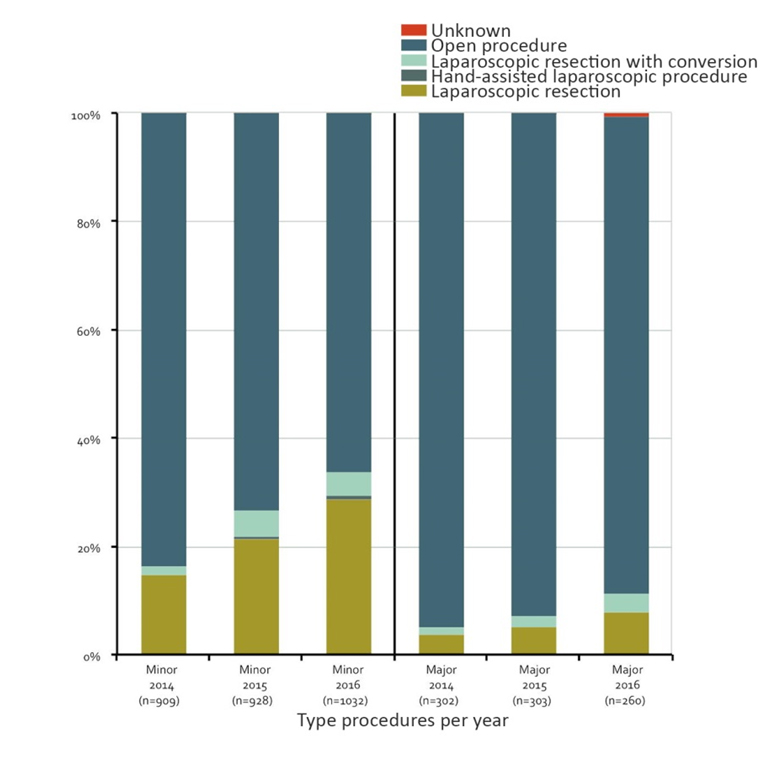

Supplement: Supplementary file 1 — Fig. S1. Division of all liver procedures for malignancies, performed in the Netherlands between 2014 and 2016, between minor (<2 segments) and major (>2 segments) resection types. [file MP-48-2145-s005.jpg]
