# Supplementary material for: CBCT‐based navigation system for open liver surgery: Accurate guidance toward mobile and deformable targets with a semi‐rigid organ approximation and electromagnetic tracking of the liver
Source: Med Phys. 2021 Apr 1;48(5):2145–59. doi: 10.1002/mp.14825 (PMC8251891; doi:10.1002/mp.14825)
Supplement: Supplementary file 2 — Table S1. Patient inclusion criteria. [file MP-48-2145-s010.doc]

**Table S-1**. Patient inclusion criteria

| **Criteria** | **Pilot phase** | **Phase I** | **Phase II** |
| --- | --- | --- | --- |
| Age | ≥ 18 | ≥ 18 | ≥ 18 |
| Surgery type | Open liver resection | Open liver resection | Open liver resection |
| Tumor type | N/A | N/A | N/A |
| Min tumor diameter | ≥ 2 cm | ≥ 2 cm | ≥ 2 cm |
| Max tumor diameter | N/A | N/A | N/A |
| Number of tumors | ≥ 1 | ≥ 1 | ≥ 1 |
| Distance from the tumor edge to the surface of the liver | ≤ 4 cm | ≤ 4 cm | ≤ 4 cm |
| eGFR | ≥ 60 | ≥ 60 | ≥ 60 |
| Iodine allergy | No | No | No |
| Diagnostic imaging modality | MRI or CT | MRI | MRI |
| Age of the diagnostic scan on the date of the surgery | ≤ 2 months | ≤ 2 months | ≤ 4 weeks |
| Pacemaker | Not present | Not present | Not present |
| Implants of prothesis with ferromagnetic material | Not present | Not present | Not present |
