# Supplementary material for: CBCT‐based navigation system for open liver surgery: Accurate guidance toward mobile and deformable targets with a semi‐rigid organ approximation and electromagnetic tracking of the liver
Source: Med Phys. 2021 Apr 1;48(5):2145–59. doi: 10.1002/mp.14825 (PMC8251891; doi:10.1002/mp.14825)
Supplement: Supplementary file 3 — Table S2. Summary of the main navigation‐related steps of the study, as was defined at the end of the learning curve phase. [file MP-48-2145-s003.doc]

**Table S-2.** Summary of the main navigation-related steps of the study, as was defined at the end of the learning curve phase.

| **Step** | **Method** |
| --- | --- |
| Patient inclusion criteria | - Scheduled for open liver surgery - Presents of superficial (<3 cm from the surface) liver lesions with >2 cm diameter - Recent MRI or CT scan (<2 months old) - GFR level above 60 (required for administration of CT contrast agent) - Informed consent |
| Preoperative 3D model | - Automatically created from diagnostic MRI or CT scan - Contains liver, portal and hepatic veins, biliary ducts and tumor contours |
| Required electromagnetic (EM) tracking components | - EM field generator - 6 degrees of freedom (DOF) EM sensor (e.g. liver tracker) - Three external patient trackers (5 DOF, EM) - 6 DOF EM probe (e.g. sterile EM pointer of the surgeon) - EM tracking and X-ray imaging compatible operation table |
| Attachment of the liver sensor | - Advanced topical skin adhesive (Ethicon, Dermabond) combined with surgical stiches |
| Intraoperative contrast-enchased CBCT imaging protocol | - Intravenous injection of Omnipaque 300 mg I/mL - Portal venous phase without bolus triggering, but generic 73 s delay between the injection and the start of CBCT imaging   Iodine contrast injection protocol:   - 45-65 kg use of 90ml at 3.2 ml/s flow - 65-80 kg use of 115 ml at 4.0 ml/s flow - 80-100 kg use of 140 ml at 4.8 ml/s flow - >100 kg use of 170 ml at 5.4 ml/s flow |
| CBCT to diagnostic scan registration | Rigid (6 DOF), gray-value based registration with region of interest restriction on the area of the liver containing the target lesion |
| CBCT to electromagnetic field registration | - Rigid point-based registration on 4 to 5 locations (phase I) |
| Accuracy measurements | - Comparison between navigation-based to pathology results for the shortest distance between measurement points in the resection plane and the tumor |
