# Supplementary material for: CBCT‐based navigation system for open liver surgery: Accurate guidance toward mobile and deformable targets with a semi‐rigid organ approximation and electromagnetic tracking of the liver
Source: Med Phys. 2021 Apr 1;48(5):2145–59. doi: 10.1002/mp.14825 (PMC8251891; doi:10.1002/mp.14825)
Supplement: Supplementary file 4 — Table S3. Comparison of pathology‐ and CBCT‐based accuracy measurements with respect to the output of our navigation system. Measurements were performed on the same locations. [file MP-48-2145-s002.doc]

**Table S-3.** Coordinates of 5-DOF sensor locations automatically detected on extended FOV and Clinical FOV CBCT reconstruction for five test patients.

| **Case number** | **Extended FOV** | | | | | | | | | | | | | | | | | | | | | | |
| --- | --- | --- | --- | --- | --- | --- | --- | --- | --- | --- | --- | --- | --- | --- | --- | --- | --- | --- | --- | --- | --- | --- | --- |
|  | **Sensor 1** | | | **Sensor 2** | | | | **Sensor 3** | | | | **Sensor 4** | | | | **Sensor 5** | | | | **Sensor 6** | | | |
|  | **x** | **y** | **z** | **x** | **y** | **z** | | **x** | **y** | **z** | | **x** | **y** | **z** | | **x** | **y** | **z** | | **x** | **y** | | **z** |
| **1** | 8.46 | -5.22 | -20.34 | 8.61 | -2.91 | -20.34 | | 4.42 | -5.29 | -19.65 | | -2.16 | -5.44 | -19.20 | | 3.24 | -9.00 | -18.93 | | 5.43 | -8.91 | -19.54 | |
| **2** | 7.31 | -3.88 | -23.74 | 6.06 | -5.75 | -23.33 | | 2.21 | -4.05 | -22.27 | | 2.11 | -1.81 | -22.29 | | -0.16 | -4.03 | -19.40 | | -0.73 | -5.41 | -17.66 | |
| **3** | 6.18 | -3.28 | -21.90 | 5.01 | -5.17 | -21.23 | | 2.44 | -2.43 | -19.35 | | 2.40 | -4.75 | -19.08 | | -0.09 | -2.53 | -18.17 | | -0.54 | -4.56 | -17.17 | |
| **4** | 5.70 | -1.37 | -21.09 | 5.48 | -3.65 | -20.65 | | 2.56 | -3.53 | -19.09 | | 1.75 | -1.55 | -19.91 | |  |  |  | |  |  | |  |
| **5** | 8.20 | -4.11 | -24.40 | 8.57 | -1.89 | -24.70 | | 4.51 | -1.70 | -23.80 | | 2.72 | -3.07 | -23.00 | |  |  |  | |  |  | |  |
| **Clinical FOV** | | | | | | | | | | | | | | | | | | | | | | | |
| **Case number** | **Sensor 1** | | | **Sensor 2** | | | | **Sensor 3** | | | | **Sensor 4** | | | | **Sensor 5** | | | | **Sensor 6** | | | |
|  | **x** | **y** | **z** | **x** | **y** | **z** | | **x** | **y** | **z** | | **x** | **y** | **z** | | **x** | **y** | **z** | | **x** | **y** | | **z** |
| **1** | 8.46 | -5.22 | -20.34 | 8.61 | -2.92 | -20.51 | | 4.41 | -5.28 | -19.65 | | 2.12 | -5.38 | -19.18 | | 3.23 | -9.02 | -18.93 | | 5.44 | -8.92 | | -19.54 |
| **2** | 7.33 | -3.89 | -23.75 | 6.09 | -5.75 | -23.33 | | n/a* | | | | n/a | | | | n/a | | | | n/a | | | |
| **3** | n/a | | | n/a | | | | n/a | | | | n/a | | | | n/a | | | | n/a | | | |
| **4** | 5.69 | -1.38 | -21.09 | 5.47 | -3.66 | -20.65 | | 2.56 | -3.54 | -19.10 | | n/a | | | | n/a | | | | n/a | | | |
| **5** | 8.22 | -4.12 | -24.35 | n/a | | | | n/a | | | | n/a | | | | n/a | | | | n/a | | | |
| **Displacement between extended FOV and clinical FOV locations** | | | | | | | | | | | | | | | | | | | | | | | |
|  | | | **Case1** | | | | **Case 2** | | | | **Case 3** | | | | **Case 4** | | | | **Case 5** | | | | |
| 3D displacement [mm] | | | 0.71 | | | | 0.02 | | | | n/a | | | | 0.01 | | | | 0.06 | | | | |
| **Average error [mm]:** | | | **0.20** | | | | | | | | | | | | | | | | | |  | |  |

*outside of the FOV

Within Phase II of the study, extended field-of-view (FOV) reconstruction of intraoperative CBCT data was used. This reconstruction involves not fully sampled projection data that extend beyond clinical FOV of Ø 25 cm, up to the edges to 35 x 35 x 20 cm image volume. Extended FOV CBCT data was used for visualization of six 5 degrees-of-freedom (DOF) electromagnetic patient trackers, which were located on the back of the patient. Center of each external patient tracker was automatically detected using in-house developed navigation software. Use of under sampled data can result in geometric deformations in extended FOV CBCT, what can cause additional inaccuracies in localization of 5-DOF EM-sensors. To assess the magnitude of this inaccuracy, absolute difference in location of the 5-DOF sensor detected on extended and clinical FOV reconstruction of the same patient were measured. In Table S3 one can see an overview of these results for five test cases. The average error stayed below 0.2 mm of 3D displacement, what is beyond EM-tracking accuracy. Therefore, possible geometric deformations of extended FOV reconstruction were considered to be negligible.
