# Supplementary material for: CBCT‐based navigation system for open liver surgery: Accurate guidance toward mobile and deformable targets with a semi‐rigid organ approximation and electromagnetic tracking of the liver
Source: Med Phys. 2021 Apr 1;48(5):2145–59. doi: 10.1002/mp.14825 (PMC8251891; doi:10.1002/mp.14825)
Supplement: Supplementary file 5 — Table S4. CBCT‐based accuracy measurement of three independent observers. [file MP-48-2145-s006.doc]

**Table S-4**. Comparison of pathology- and CBCT- based accuracy measurements with respect to the output of our navigation system. Measurements were performed on the same locations.

| **Test case** | **Measurement point 1** | | | **Measurement point 2** | | | **Measurement point 3** | | | **Average error [mm]** | |
| --- | --- | --- | --- | --- | --- | --- | --- | --- | --- | --- | --- |
|  | **Pathology** | **CBCT** | **Navigation** | **Pathology** | **CBCT** | **Navigation** | **Pathology** | **CBCT** | **Navigation** | **Pathology-to-Navigation** | **CBCT-to-Navigation** |
| 1 | 74 | 58 | 53 | 59 | 56 | 56 | 40 | 56 | 52 | 24.4 | 6.4 |
| 2 | 11 | 24 | 22 | 8 | 21 | 21 | 26 | 6 | 3 | 28.6 | 3.6 |
| 3 | 10 | 16 | 25 | 23 | 34 | 34 | 17 | 16 | 21 | 19.0 | 10.3 |
| 4 | 10 | 21 | 26 | 29 | 26 | 26 | 37 | 50 | 33 | 16.8 | 17.7 |
