# Supplementary material for: CBCT‐based navigation system for open liver surgery: Accurate guidance toward mobile and deformable targets with a semi‐rigid organ approximation and electromagnetic tracking of the liver
Source: Med Phys. 2021 Apr 1;48(5):2145–59. doi: 10.1002/mp.14825 (PMC8251891; doi:10.1002/mp.14825)
Supplement: Supplementary file 6 — Table S5. CBCT‐based accuracy measurement of three independent observers. [file MP-48-2145-s009.doc]

**Table S-5.** CBCT-based accuracy measurement of three independent observers.

| **Measurement location** | **Measurement** | **Patient number** | | | | | | | | |
| --- | --- | --- | --- | --- | --- | --- | --- | --- | --- | --- |
|  |  | **26** | **27** | **28** | **29** | **30** | **32** | **33** | **34** | **35** |
| Point 1 | Error [mm] | 3,35 ±0,39* | 0,54 ± 7,31 | 4,98 ± 1,03 | -1,44± 3,49 | -6,99± 1,68 | -2,80± 2,10 | 10,91±4,36 | 1,30± 1,78 | -1,68±6,67 |
| Point 2 | Error [mm] | 3,59 ± 2,62 | -1,9 ± 7,05 | 1,24 ± 4,63 | -2,10 ± 2,57 | -10,59 ± 5,51 | -2,21 ± 4,91 | -5,49 ± 0,48 | -8,48 ± 1,85 | -7,7 ± 6,63 |
| Point 3 | Error [mm] | 3,52 ± 5,35 | -2,95 ± 6,49 | -0,94 ± 0,66 | 1,13 ± 5,02 | -7,13 ± 1,92 | -2,40 ± 1,71 | 8,49 ± 2,68 | 2,95 ± 5,47 | 4,45 ± 0,93 |
| Point 4 | Error [mm] | -2,11 ± 3,90 | 6,93 ± 6,92 | -3,43 ± 1,57 | 2,47 ± 4,53 | -6,55 ± 1,93 | -1,72 ± 5,76 | 1,72 ± 2,39 | -9,88 ± 1,06 | 1,22 ± 1,95 |
| Point 5 | Error [mm] |  |  | 1,05 ± 0,05 |  | -8,62 ± 3,96 |  | 1,93 ± 2,88 | 3,30 ± 1,48 |  |

*standard deviation over measurement of three observer

In phase II of the study, intraoperative accuracy measurements (i.e., based on the navigation software) were compared with the corresponding distance measured on CBCT images, to determine the absolute accuracy of the technique. Postoperative CBCT-based measurements were performed by three independent expert users (Table S-5). The average value of the three observers was used to determine the accuracy of the system.
