# Supplementary material for: CBCT‐based navigation system for open liver surgery: Accurate guidance toward mobile and deformable targets with a semi‐rigid organ approximation and electromagnetic tracking of the liver
Source: Med Phys. 2021 Apr 1;48(5):2145–59. doi: 10.1002/mp.14825 (PMC8251891; doi:10.1002/mp.14825)
Supplement: Supplementary file 11 — Data S1. Study design and sample size calculation. [file MP-48-2145-s011.doc]

**Supporting Information file**

## Study design and sample size calculation

Because the learning curve corresponds to the initial feasibility phase of the study, no statistic sample size calculation was used, and this stage corresponded to the first 10 patients within the study. For two following phases, the minimal study sample calculation was performed using “1 Proportion: 1-Sample, 1-Sided test” (H0 (p = p0) and H1 (p < p0)). All calculations were performed with an α = 0.05 and β = 0.2. Expected success rate of the procedures was set to 85% (P), based on the results of the navigation study on semi-rigid targets described in Nijkamp et al. ^21^. However, considering higher mobility of the organ in the current setup, P0 was set to 65%. As a result, a minimum of 30 individual accuracy measurements per phase were required to reach the significance level, with the acceptable lover limit of two and an upper limit of five accuracy measurements per patient. In total, no more than 53 patient were allowed to be included in the study (e.g., 10 – learning curve, and 43 cases in the following two phases).
